# Antibacterial Effect of Calcium Hydroxide on Intraluminal and Intratubular Enterococcus Faecalis

**Published:** 2011-08-15

**Authors:** Maryam Javidi, Mina Zarei, Farzaneh Afkhami

**Affiliations:** 1. Department of Endodontics, Dental Material Research Center, Mashad, Iran.; 2. Department of Endodontics,Mashad University of Medical Sciences, Mashad, Iran.; 3. Department of Endodontics, Dental School, Masshad University of Medical Sciences, Mashad, Iran.

**Keywords:** Calcium Hydroxide, Enterococcus Faecalis, Medicaments, Root Canal Treatment

## Abstract

**INTRODUCTION:**

Root canal treatment involves the elimination of intraradicular microorganisms. Calcium hydroxide [Ca(OH)_2_] is the most widely used canal dressing material. Enterococcus faecalis (E. faecalis) has been reported to be resistant to Ca(OH)_2_ in-vivo. The purpose of this study was to evaluate the efficacy of Ca(OH)_2_ on the elimination of intraluminal and intratubular E. faecalis.

**MATERIALS AND METHODS:**

Thirty six human single-rooted teeth were contaminated with E. faecalis. Thirty specimens in the experimental group were treated with 10% Ca(OH)_2_; six specimens were treated with normal saline as the positive control (n=6). Specimens from experimental group were randomly divided into two subgroups of 15 each. In subgroup A, specimens were incubated and sampled after one day and in subgroup B, they were tested at day seven. Paper points and Gates Glidden burs were used to obtain the intraluminal and intratubular E. faecalis respectively. Samples obtained from these root canal preparations were analyzed for bacterial load by counting the number of colony forming units (CFUs). Mann-Whitney and t-test were used for analysis.

**RESULTS:**

Group B had significant decrease in CFUs compared with group A with both sampling methods (P<0.001). No differences were observed between the antimicrobial properties of Ca(OH)_2_ against intraluminal and intratubular E. faecalis. After 1 week, there was a significant reduction in CFU load with Ca(OH)_2_ intra canal medication.

**CONCLUSION:**

Ca(OH)_2_ showed the same antimicrobial efficacy on intraluminal and intratubular E. faecalis.

## INTRODUCTION

The bacteria and their byproducts play a major role in the development of pulpal and periapical diseases [1]. The main objective of root canal therapy is to eliminate bacteria from infected root canal system to prevent reinfection and assist healing [2]. Because of the complex anatomy of the root canal system such as lateral and accessory root canals, isthmi, and apical rims and ramifications, microorganisms can escape from chemomechanical debridement [3].

Enterococcus faecalis (E. faecalis) is a facultative gram-positive bacterium which can remain in the root canals and cause refractory or persistent periapical diseases [4][5]. This bacterium can adhere to dentin collagen (main organic component of dentine), invade the dentinal tubules and therefore withstand root canal debridement [6]. Furthermore, E. faecalis can tolerate high alkalinity due to its proton pumps [7].

Among the intracanal disinfectants, calcium hydroxide [Ca(OH)_2_] is the most commonly used medicament due to its good properties such as wide antimicrobial spectrum, alkalinity, induction of hard tissue formation, and anti-inflammatory effects [8]. However, several studies have attested the inefficacy of calcium hydroxide in eliminating E. faecalis, which is often isolated in persistent infections of root canal system [9][10][11].

The purpose of this in vitro study was to test the antibacterial efficacy of Ca(OH)_2_ against intraluminal and intratubular E. faecalis.

## MATERIALS AND METHODS

Thirty-six extracted single-rooted human teeth were decoronated to a standard 15mm root length. Each root canal was prepared using RaCe rotary system according to manufacture instructions. RC Prep (Premier Dental Products, Norristown, PA, USA) was used as lubricant, and canals were irrigated with 2.5% sodium hypochlorite (NaOCl) throughout instrumentation. The canals were irrigated with 1mL of 17% EDTA solution and then 1mL of 5.25% NaOCl. The final canal irrigation consisted of 10mL physiologic saline solution. Teeth were subsequently autoclaved at 121°C for 30 minutes. Two layers of nail polish were carefully applied over external root surfaces keeping away from the root canal entrance to avoid external microbial contamination.

Root specimens were transferred into sterile microcentrifuge tubes under sterile conditions. Then, 1mL of BHI broth containing 1.5×10^8^ bacterial cells (E. faecalis; ATCC 29212) from a micro-tube of #0.5 McFarland was injected into the prepared root canal system using a 30-gauge irrigation needle. After injection, each specimen was entirely submerged in BHI broth, and the tubes were incubated an aerobically at 37°C for 21 days. After incubation, the medium was aseptic-ally aspirated from the tubes and the canals were blotted dry with sterile paper points. The specim-ens were randomly divided into two experimental groups (n=30) and a negative control group (n=6). The experimental group was further divided in groups A and B (n=15). Control group was also divided into A and B (n=3).

Groups A and B specimens of the experimental batch were submitted to intracanal dressings. A mixture of Ca(OH)_2_ (10% concentration) was used consisting of 0.1g Ca(OH)_2_ (Golchadnet, Iran) per 1.0mL sterile water. Under aseptic conditions, the canals of the experimental groups were filled with medicament using a sterile 3-mL syringe and 27-gauge needle until the canals were totally filled. In the negative control group with inoculated specimens, the teeth received sterile water only.

Group A samples of experimental and control groups were incubated at 37°C for 1 day and group B samples were incubated at 37°C for 1 week.

At the end of incubation, the temporary filling was removed, and the root canals were rinsed with 10mL of sterile saline. Subsequently, root canals were irrigated with 0.5% citric acid which is a neutralizing solution for Ca(OH)_2_. A last irrigation with 10mL sterile saline was then performed.

The first bacterial sampling was taken using sterile paper point size 50 (Excel Dental Supplies Ltd., Hong Kong) that was placed inside the root canal for 1 minute. Paper point was placed in a microcentrifuge tubes containing 1mL of BHI broth and shaken for 30s on a Vortex mixer (v-1 BOECO, Germany). Then, 0.1mL of the microbial suspension was placed on BHI agar plates and incubated at 37°C for 24 hours.

After sampling with paper points, Gates Glidden burs #5 (Dentsply, Maillefer, Switzerland) and Endo IT electromotor (VDW, Germany) at 2000 rpm were used to attain samples from dentin. Each bur was used once up to 10mm of canal length. After the purity confirmation, the colonies on the agar plates were counted.

The SPSS computer software version 16.0 was used to conduct data analysis. Descriptive statistics including means, standard deviations and frequency distribution were calculated for each subgroup. The Mann-Whitney and t-test were used to compare the antimicrobial activity of the test medications and Wilcoxon t-test was used to compare these two methods. Values of P<0.05 were considered statistically significant.

## RESULTS

The antimicrobial activity of Ca(OH)_2_ was evaluated by counting the CFUs. The results of intratubular and intraluminal samples were not statistically different in each period of study (P>0.05) (Table 1). After 1 and 7 days of intracanal medication, treatment with sterile water did not influence the bacteria viability within the lumen or dentinal tubules and the number of CFUs of E. faecalis was >1000 colony per control agar plate.

**Table 1:** Statistical Indices of CFUs of E. faecalis (Mean±SD) for canals treated with Ca(OH)_2_

**Time**	**Sampling method**	
	**Paper Point**	**Gates Glidden**
**1 day**	550±120	623±154
**1 week**	92.6±8.2	75.3±5.2

Compared with control group (sterile water), Ca(OH)_2_ reduced CFUs of E. faecalis which was recovered after each period of study.

The treatment with Ca(OH)_2_ presented significantly less number of CFUs after 7 days compare to 1 day (P<0.001) regardless of method of sampling.

## DISCUSSION

Although chemomechanical preparation reduces microbiota effectively, these procedures do not provide complete bacteria elimination; this is due to the complex anatomy of the root canal system and the persistent microorganisms [11].

E. faecalis is a gram-positive bacterium which is often isolated in persistent apical infections as it is more challenging to eradicate [12]. Even though Ca(OH)_2_ has good antimicrobial properties, it was not capable of thoroughly destroying E. faecalis under the conditions of the present study. This is in accordance with the finding of other reports [13][14][15]. The antimicrobial efficacy of Ca(OH)_2_ is related to the release of hydroxide ions in an aqueous environment and so depends on the concentration of hydroxide ions in the solution; therefore, thick mixtures of Ca(OH)_2_, may not be ideal for use as an intracanal dressing [16]. A 10% concentration was used as suggested in other studies [16][17].

Smear layer removal has often been suggested because it can open dentinal tubules and allow penetration of liquids and microorganisms [18][19]. Hence, EDTA and subsequently, NaOCl irrigation was used.

The time needed for Ca(OH)_2_ to achieve an optimal antibacterial effect has not been fully established [20]. Kayaoglu et al. [21] demonstrated elimination of E. faecalis from infected dentine specimens after 1 day of exposure; while, Sjögren et al. [22] claim that Ca(OH)_2_ must remain in the canal for 1 week. In this study Ca(OH)_2_ was not able to eliminate E. faecalis thoroughly even after 1 week; however, it significantly reduced this bacterium.

In the current in vitro study, microbiological sampling was accomplished using two methods consist of sterile paper point that absorbed the planktonic microorganisms suspended at the lumen of the root canal and Gates Glidden burs that was allowed the recovery of bacteria inside dentinal tubules [19][22]. No difference was observed between the two in our study.

## CONCLUSION

Ca(OH)_2_ showed the same antimicrobial efficacy on intraluminal and intratubular E. faecalis, although it was unable to thoroughly eliminate E. faecalis from root canals.
